# Optical Transitions and Excited State Absorption Cross Sections of SrLaGaO_4_ Doped with Ho^3+^ Ions

**DOI:** 10.3390/ma14143831

**Published:** 2021-07-08

**Authors:** Marcin Kaczkan, Michał Malinowski

**Affiliations:** Institute of Microelectronics and Optoelectronics, Warsaw University of Technology, Koszykowa 75, 00-662 Warsaw, Poland; m.malinowski@elka.pw.edu.pl

**Keywords:** holmium, luminescence, SrLaGaO_4_:Ho^3+^, excited state absorption (ESA)

## Abstract

The spectroscopic properties of SrLaGaO_4_ (SLO) crystal doped with Ho^3+^ ions were studied in this work. Absorption, emission spectra and decay dynamics of excited states have been measured and discussed using the Judd–Ofelt model. Photoluminescence emissions were attributed to transitions from the excited ^3^D_3_, ^5^S_2_, ^5^F_5_, ^5^I_6_ and ^5^I_7_ multiplet manifolds. The experimental lifetimes for five excited states have been compared to the theoretical values, calculated using Judd–Ofelt theory, allowing for the determination of the multiphonon relaxation rates (W_nR_) of the respective states. The experimental data were approximately on a line expressed by W_nR_ = W_0_ exp(−αΔE) with W_0_ = 0.5 × 10^7^ s^−1^ and α = 2.6 × 10^−3^ cm. To discuss the excited state absorption (ESA) pathways, that originated from several excited levels, we used the Judd–Ofelt formalism allowing determination of the integrated cross section for ESA transitions.

## 1. Introduction

The strontium lanthanum gallate SrLaGaO_4_ (SLO) and its solid solutions have been extensively investigated since they were considered as a promising substrate for high temperature superconducting (HTSc) films [1]. SLO doped with transition metal (TM) or rare earth (RE) ions was also considered as laser active media [2]. Recently, SLO has been investigated as a host lattice for phosphors, which exhibit unusual and interesting properties when activated by RE or TM ions [3,4]. In particular, when Tb^3+^ is introduced into SrLaGaO_4_, long persistent green emission has been reported [3]. The duration of green afterglow was observed even after more than 3.5 h. A novel, far-red emitting SrLaGaO_4_:Mn^4+^ phosphor with high brightness and excellent luminescent properties has been synthesized by the simple solid-state reaction and investigated in [5]. In addition, the luminescence spectra of SLO activated by Cr^3+^ were reported in [4]. It was shown that Cr^3+^ ions occupy more than four different sites in the SLO lattice, which is related to the random distribution of Sr^2+^ and La^3+^ ions.

The interest in studying RE^3+^ doped SLO strongly results both from its structural disorder, and the resulting inhomogeneous broadening of the optical transitions [6], as well as from the ability of this matrix to accept high concentrations of activator [1,2]. Such a broadening helps the generation of ultrashort pulses by mode locking. Thus, due to its good thermal properties and broad gain spectrum, Yb^3+^-doped strontium lanthanum aluminate crystal was reported as a promising material for power-scalable, broadly tunable CW and sub-100 fs mode-locked lasers at ~1 μm [7].

Holmium ion activated solids have been the subject of extensive investigations for the development of laser materials, phosphors and color displays [8,9,10]. The use of Ho^3+^ as a potential candidate for temperature sensing has been confirmed, and thermal-coupled levels of Ho^3+^ ions, ^5^F_4_ and ^5^S_2_, ^5^S_2_ and ^5^F_5_, have been investigated in temperature measurement applications [11].

In particular, much attention has been dedicated to the studies of up-conversion processes in Ho^3+^ doped low-energy phonon crystals, optical glasses [12] and glass ceramics [13]. Up-conversion materials have wide applications, such as for bio-imaging, photo-therapies, photo-catalysis, solar conversion, temperature sensing, etc. Generally, energy transfer up-conversion (ETU), excited state absorption (ESA), and photon avalanche (PA) are the three main channels for up-conversion fluorescence. The Ho^3+^ ion is very suitable for use in up-conversion processes because it has many long-lived intermediate metastable levels, from which ESA can take place resulting in a strong, green anti-Stokes luminescence from ^5^S_2_ level which has been demonstrated in a number of Ho^3+^ doped materials after red and infrared excitation [14]. It was also demonstrated that ESA has an important influence on the performance of Ho-doped fiber lasers [15]. Thus, for the modeling of these up-conversion excitation processes the ESA cross sections have to be known.

**Figure 1:**
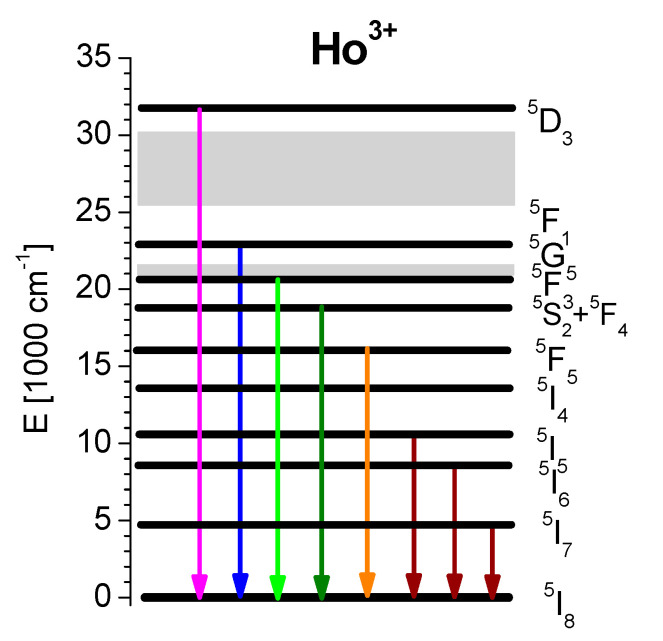
The partial energy diagram of Ho^3+^ ions in SrLaGO_4_ crystal. Arrows indicate the emission transitions.

Although the optical properties of Nd^3+^, Pr^3+^, Eu^3+^, Yb^3+^ and Tm^3+^ doped SLO crystals have been reported [3,6,7,16], the spectroscopic data on Ho^3+^ doped SLO have not, to our knowledge, been presented in detail. In [17] we reported on the nature of strong spectral lines broadening in Ho^3+^ doped SrLaGa_3_O_7_ and SrLaGaO_4_ crystals which have been investigated by using the non-resonant fluorescence line narrowing (FLN) technique. Also, up-conversion ultraviolet emission from ^3^D_3_ level in these compounds was observed in [18]. The partial energy diagram of Ho^3+^ ions in the SLO host, together with the strongest observed emission transitions, is illustrated in Figure 1.

Recently, we studied ESA processes that involve the ^5^I_6_ and ^5^I_7_ energy levels of Ho^3+^ in singly Ho^3+^-doped ZrF_4_–BaF_2_–LaF_3_–AlF_3_–NaF (ZBLAN) glass [12] and also YAlO_3_ (YAP), Y_3_Al_5_O_12_ (YAG), LiYF_4_ (YLF) and SrLaGa_3_O_7_ (SLG) crystals [14]. In the present investigation, we extend this work by identifying and quantifying a range of pump excited state absorption ESA processes in SLO that may affect the functioning of Ho^3+^-doped SLO green phosphor or laser.

## 2. Materials and Experiment

A SrLaGaO_4_:Ho^3+^ crystal with activator concentrations of 0.3 at % was grown using the Czochralski technique at ITME Laboratory in Warsaw. The samples used were monocrystalline of good optical quality which, after orientation, were cut into 10 × 5 × 2 mm^3^ plates and polished carefully in order to meet the requirements for spectroscopic measurements. Polarized absorption spectra were measured in the 200–2900 nm range of wavelength at room temperature on a Cary-50 UV–Vis-NIR spectrophotometer (Varian, Inc., Palo Alto, CA, USA) equipped with Glan–Taylor polarizers.

Luminescence from the sample was excited using both pulsed and CW laser sources. For the pulsed measurements an optical parametric oscillator Surelite OPO (Continuum, Santa Clara, CA, USA) with YAG:Nd laser (Surelite II, Continuum Santa Clara, CA, USA) as a pumping source was used. CW excitation was performed with tunable Ti-sapphire laser (Coherent 899 Ring Laser, (Santa Clara, CA, USA)) pumped by argon laser (Coherent Innova 300, (Santa Clara, CA, USA). Optical signal from the sample was analyzed using DK480 monochromator (CVI Laser Corporation, Albuquerque, NM, USA) and detected by cooled photomultiplier tubes (EMI C1034-02 GaAs (EMI Electronics Ltd., London, England) or Hamamatsu 7102 (Hamamatsu Photonics K.K., Hamamatsu, Japan)) and PbS detector for visible and infrared range, respectively. Stanford SR 400 photon-counting system (Stanford Research Systems Sunnyvale, CA, USA) controlled by PC was used to perform data acquisition. Measurements of luminescence decays were recorded using Stanford SR 430 multichannel analyzer (Stanford Research Systems Sunnyvale, California, USA). Low temperatures of the samples were obtained with a Displex Model CSW-202 cryostat (Apd Cryogenics Inc., Allentown, PA, USA).

**Figure 2:**
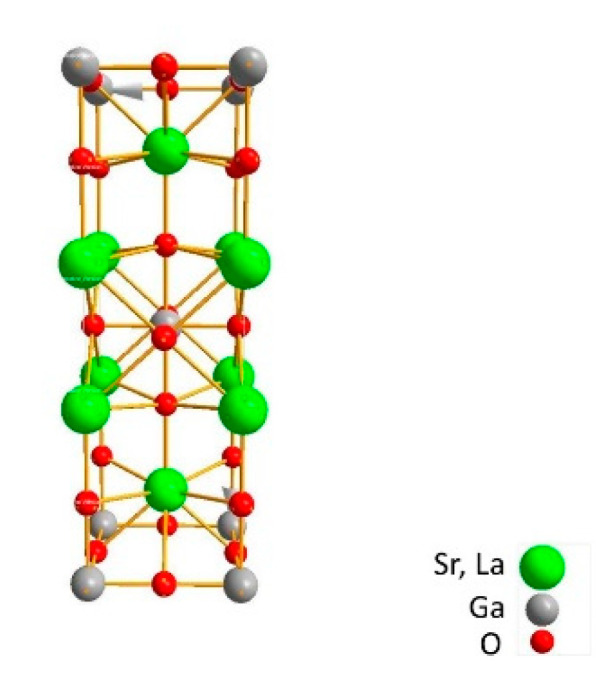
Schematic crystal structure of SrLaGaO4.

SrLaGaO_4_ belongs to a large family of compounds of general chemical formula ABCO_4_ where A = Ca, Sr, Ba; B = Y, La-Gd; C = Al, Ga. SLO crystallizes in a perovskite-like, tetragonal structure, space group I4/mmm with unit cell parameters of a = b = 0.38437(3) nm and c = 1.26880(15) nm, the volume of the unit-cell V = 178.27(1) Å^3^ and the number of structural units Z = 2 [19,20]. The crystal structure is built from CO_6_ (GaO_6_) layers formed in the ab plane. The Sr^2+^ and La^3+^ cations are distributed randomly between the layers, taking positions in nine coordinated sites with distorted C4v symmetry. The C cations have a coordination of six and are in a slightly distorted octahedral environment, while Sr and La ions are surrounded by nine oxygens. The elementary cell of SrLaGaO_4_ is shown in Figure 2 with the crystal built along the c-axis.

Since the radius of the Ho^3+^ ion (0.172 Å) is comparable to those of the La^3+^ (0.126 Å) and Sr^2+^ (0.131 Å), it can be assumed that both La^3+^ and Sr^2+^ sites can be occupied by Ho^3+^ in SrLaGaO_4_. It has been shown that the random distribution of the A and B cations over different lattice positions leads to a disordered structure of these crystals resulting in the strong inhomogeneous broadening of the spectral lines.

## 3. Results and Discussion

### 3.1. Absorption Spectra and Optical Transition Intensity Analysis

**Figure 3:**
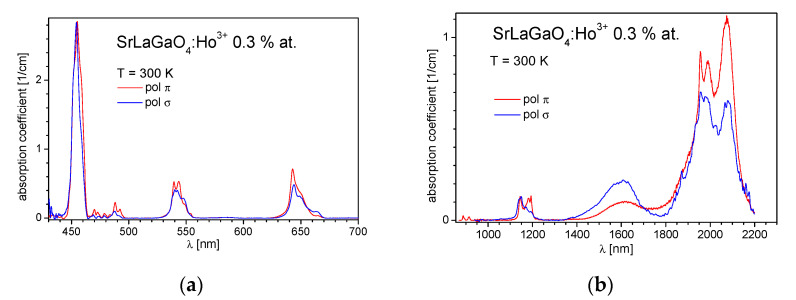
(**a**) Visible and (**b**) Infrared part of the polarized absorption spectrum of SrLaGaO_4_:Ho^3+^ crystal recorded at room temperature.

The structure makes SrLaGaO_4_ uniaxially anisotropic where the strength of the absorption lines is dependent on the direction of the incident light. Bearing in mind that the optical axis is parallel to the crystallographic c-axis, two orthogonal light polarizations, π (E || c) and σ (E ⊥ c), should be taken into account. Thus, in our spectroscopic study, we have measured the absorption characteristics for two principal light polarizations for a-cut 0.3 at% Ho^3+^ doped SrLaGaO_4_ crystal. The measured spectra in the 300–2200 nm range are presented in Figure 3.

Once the polarized absorption spectra had been measured, integrated absorbances Γ were calculated according to Equation (1) [21]:

(1) $$\bar{\Gamma }=\int_{band}\bar{\alpha }\left(\lambda \right)d\left(\lambda \right)=-\frac{1}{L}\int_{band}\ln[\frac{1}{3}\exp(-\alpha_{\pi }(\lambda )L)+\frac{2}{3}\exp(-\alpha_{\sigma }(\lambda )L)]d(\lambda )$$

where *L* is the distance the light has traveled inside the sample, α_π_(λ) and α_σ_(λ) are the absorption coefficients for *π* and *σ* polarizations and *λ* is wavelength. Integrated absorbances determined in this way were used to obtain the experimental line strengths needed to perform the Judd–Ofelt [22,23] intensity analysis. Because the drawbacks and precision of the Judd–Ofelt theory have been analyzed in detail elsewhere [24], only the essential results have been included in this article. The main point of this approach is that the oscillator strength *f_calc_* of the electric dipole transition between multiplets of lanthanide ion (*J* → *J’*) is described as:

(2) $$f_{calc}=\frac{8\pi^{2}mc}{3h\left(2J+1\right)}\frac{1}{\lambda }\overset{¯}{\chi }\underset{t=2,4,6}{\sum }{\left|U_{JJ^{©}}^{\left(t\right)}\right|}^{2}\Omega_{t}$$

where *h* is Planck’s constant, *J* is the angular momentum of the initial level, $\bar{\chi }=\frac{1}{3}(\chi_{\pi }+2\chi_{\sigma })$ is an average local field correction factor, where for electric dipole transition $\chi_{\pi ,\sigma }=\frac{{\left(n_{\pi ,\sigma }^{2}+2\right)}^{2}}{9n_{\pi ,\sigma }}$, *U*^(*t*)^ are the doubly reduced matrix elements and Ω*_t_* are empirically determined parameters. The value of oscillator strength can be also obtained from experimentally measured absorption or emission spectra. For the absorption line, the experimental oscillator strength *f_exp_*, can be defined as

(3) $$f_{exp}=\frac{mc^{2}\varepsilon_{0}}{\pi e^{2}N}\bar{\Gamma }$$

where *e* and *m* are the electron charge and mass respectively, $\bar{\Gamma }$ is the integrated absorbance obtained from Equation (1) and *c* represents the light velocity.

From the least-square fit of calculated (*f_calc_*) and measured (*f_exp_*) oscillator strengths the three Ω*_t_* intensity parameters were obtained. The reduced matrix elements needed for this calculation were taken from [25]. The values of calculated and measured oscillator strengths together with the average wavelengths for all measured absorption lines are presented in Table 1. The Judd–Ofelt intensity parameters were evaluated to be Ω_2_ = 1.25 × 10^−20^ cm^−1^, Ω_4_ = 0.42 × 10^−20^ cm^−1^, Ω_6_ = 1.80 × 10^−20^ cm^−1^ with RMS deviation between the calculated and measured oscillator strength values of 5.3 × 10^−6^. The obtained measure of the quality of the fit is closed to values found by applying the Judd–Ofeld approach to other materials doped with holmium ions [26].

Comparison of Judd–Ofelt parameters between Ho^3+^:SLO and other Ho^3+^—doped hosts is shown in Table 2.

From the calculated set of Ω*_t_* intensity parameters the electric dipole transition probabilities A_JJ’_ for emissions between J and J’ manifolds of Ho^3+^ were calculated using the equation:

(4) $$A_{JJ^{’}}=\frac{64\pi^{4}e^{2}}{3h(2J+1)}\frac{1}{\lambda^{3}}\bar{\chi }{\sum_{t=2,4,6}\left|U_{JJ^{n}}^{\left(t\right)}\right|}^{2}\Omega_{t}$$

Evaluated probabilities *A_JJ’_* of the radiative transitions from the excited states, the branching ratios *β_calc_* and resulting radiative lifetimes are presented in Table 3. The values of radiative lifetimes of the ^3^D_3_, ^5^F_3_, ^5^S_2_, ^5^F_5_, ^5^I_4_, ^5^I_5_, ^5^I_6_ and ^5^I_7_ were calculated to be: 477 μs, 217 μs, 211 μs, 372 μs, 5.34 ms, 3.37 ms, 2.83 ms and 8.02 ms, respectively.

**Figure 4:**
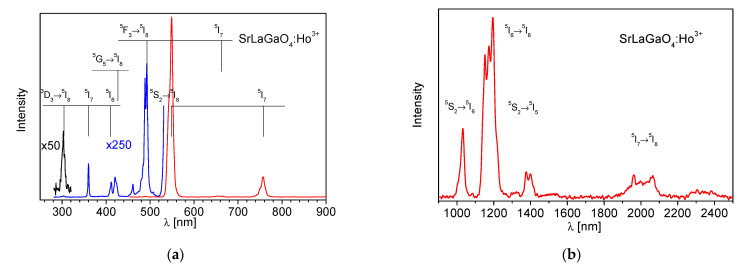
(**a**) UV-Vis and (**b**) IR part of polarized emission spectrum of SrLaGaO_4_: Ho^3+^ recorded at room temperature.

The emission characteristic of the crystal was measured at room temperature in the 250 nm–2.5 μm spectral range after CW excitation. All experimentally observed emission lines corresponding to transitions from the ^3^D_3_, ^5^F_3_, ^5^S_2_, ^5^F_5_, ^5^I_6_ and ^5^I_7_ excited states of SrLaGaO_4_:Ho^3+^ are presented in Figure 4. The emission spectrum is dominated by ^5^S_2_ → ^5^I_8_ transition, resulting in the strongest luminescence line at about 550 nm.

### 3.2. Luminescence Dynamics

**Figure 5:**
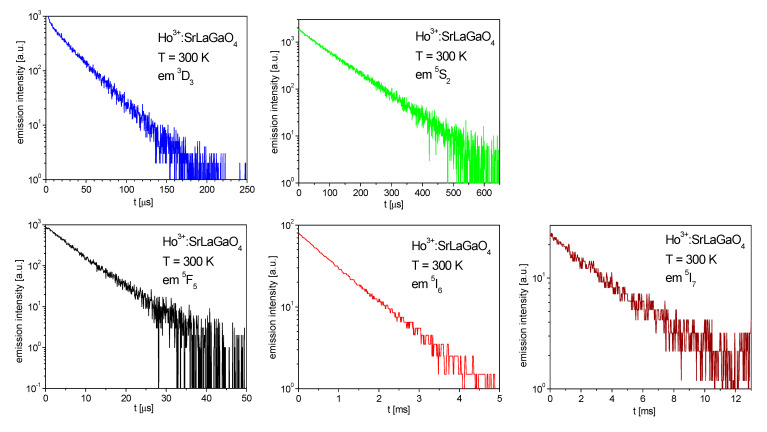
Decay profiles of the ^3^D_3_, ^5^S_2_, ^5^F_5_, ^5^I_6_ and ^5^I_7_ emission in Ho^3+^ doped SrLaGaO_4_ crystal measured at 300 K.

Fluorescence decay curves were registered after wavelength selective pulsed excitation. Figure 5 shows decay profiles of the ^3^D_3_, ^5^S_2_, ^5^F_5_, ^5^I_6_ and ^5^I_7_ emissions in 0.3% Ho^3+^ doped SrLaGaO_4_ measured at 300 K. The fluorescence lifetimes of ^3^D_3_, ^5^S_2_, ^5^F_5_, ^5^I_6_ and ^5^I_7_ manifolds were determined with estimated relative errors of 1% to be of 35 μs, 98 μs, 6 μs, 1 ms and 4 ms, respectively. Due to the large energy gap to the next lying lower level of about 4000 cm^−1^ the ^5^I_6_ and ^5^I_7_ decays could be considered as being predominantly radiative which was confirmed by its temperature independence.

Knowing values of radiative and measured lifetimes, the rates of multiphonon relaxations can be simply calculated using the equation:

(5) $$W_{nr}=\frac{1}{\tau_{obs}}-\frac{1}{\tau_{R}}$$

Calculated radiative lifetimes, measured at low temperature lifetimes, the energy gaps to the next lower level and the multiphonon transition rate for excited states of Ho^3+^ in SrLaGaO_4_ are shown in Table 4.

Dependence of the multiphonon transition probability *W_nr_* on the energy gap to the next lower level Δ*E* can be described as an exponential function [30]:

*W_nr_* = *W*_0_ × *exp*(−αΔ*E*) (6)

**Figure 6:**
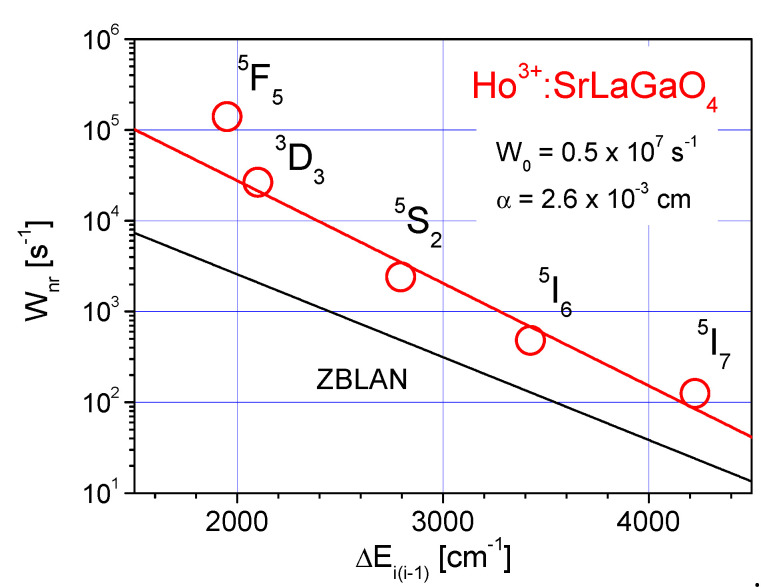
The multiphonon relaxation rate W_nr_ as a function of energy gap ΔE for the excited states of Ho^3+^ ion in SrLaGaO_4_—red line. Open circles denote experimental points. For comparison the data for ZBLAN glass are also presented—blue solid line.

The *W_nr_*(Δ*E*) dependence, together with the data for low phonon ZBLAN glass [31], is shown in Figure 6. The values of *W*_0_ and *α* in Equation (6) were calculated as 0.5 × 10^7^ s^−1^ and 2.6 × 10^−3^ cm, respectively.

As can be observed from Figure 6 the nonradiative decay rates in SLO are about one order of magnitude higher than in ZBLAN. The difference in results from higher in the case of SLO of the the phonon frequency distribution function, which is approximately hω_max_ = 707 cm^−1^ [32], than in ZBLAN matrix, which is 580 cm^−1^ [3].

### 3.3. Excited State Absorption Transitions

The high number of long-lived multiplets present in Ho^3+^ acting as good population reservoirs leads to the possibility of ESA transitions into higher lying energy levels. The intermediate levels for ESA are therefore ^5^I_J_ states (*J* = 5, 6, 7). Using the Judd–Ofelt formalism it is possible to estimate the ESA integrated cross sections by means of the following expression:

(7) $$\sigma_{int}=\overset{\lambda 2}{\underset{\lambda 1}{\int }}\sigma (\lambda )d\lambda =\frac{8\pi^{3}e^{2}}{3hc\;\left(2J+1\right)}\chi \underset{t=2,4,6}{\sum }{\left|U_{JJ’}^{\left(t\right)}\right|}^{2}\Omega_{t}$$

where *λ* is the mean wavelength corresponding to the ESA transition.

**Figure 7:**
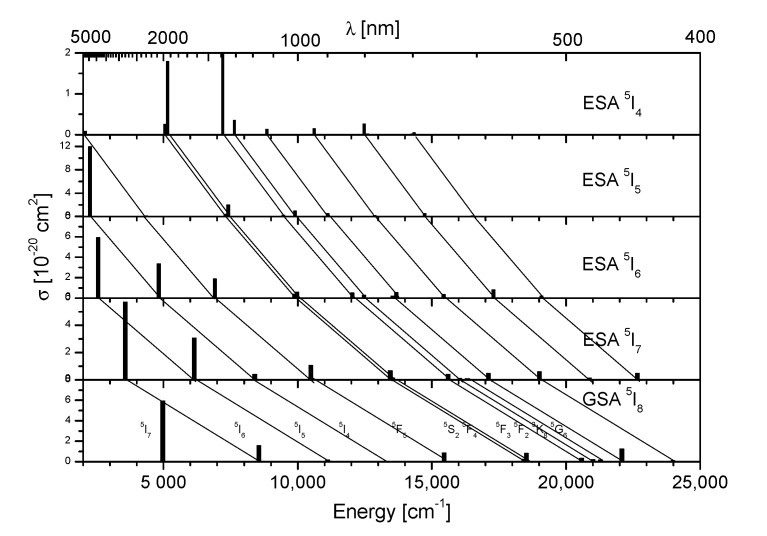
Spectrum of calculated ground state absorption (GSA) and excited state absorption (ESA) integrated cross sections σ in SrLaGaO_4_:Ho^3+^ crystal.

With this method, the ESA transition cross sections in the spectral range between 400 and 5000 nm in Ho^3+^-doped SLO were calculated and are presented in Figure 7, together with the GSA cross sections.

**Figure 8:**
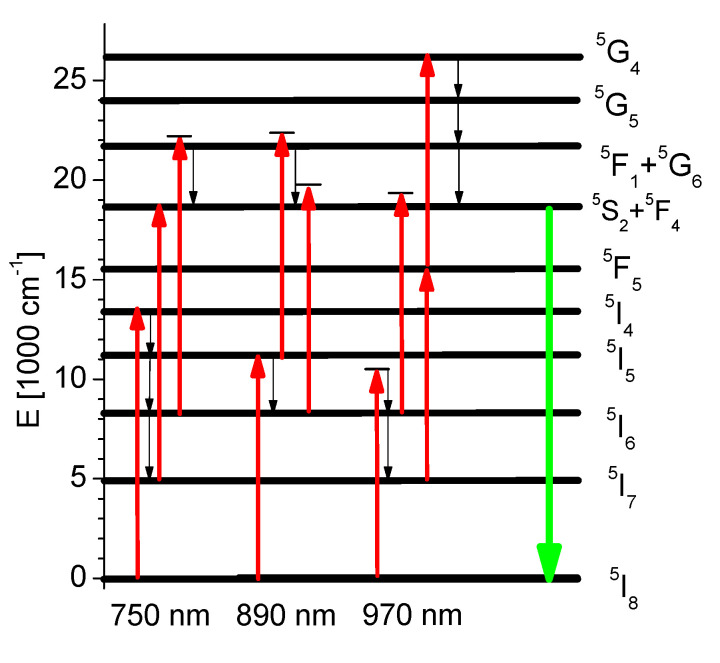
Energy level diagram of Ho^3+^ presenting different paths of populating the ^5^S_2_ state of Ho^3+^ involving GSA and ESA processes.

**Figure 9:**
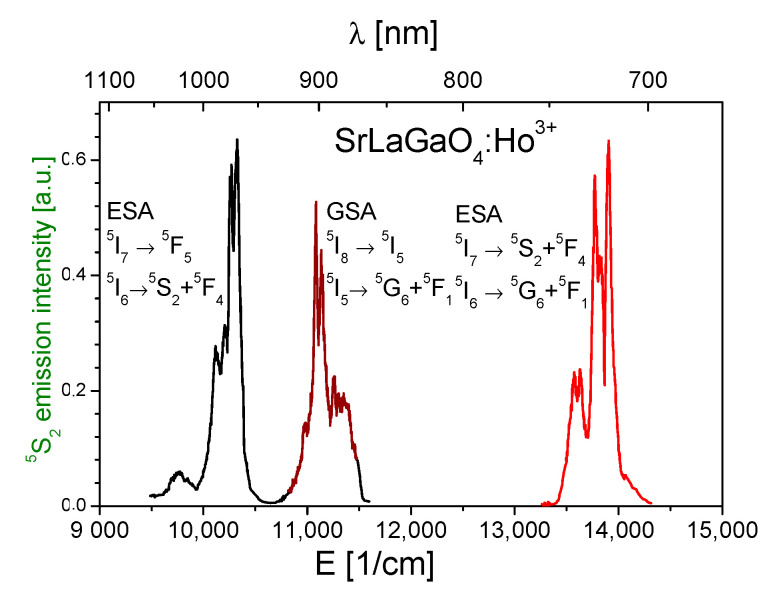
Excitation spectra of SrLaGaO_4_:Ho^3+^ crystal when monitoring the strongest ^5^S_2_ → ^5^I_8_ emission transition at 550 nm, T = 300 K.

Figure 7 also illustrates that several infrared excitation wavelengths could lead to population of the ^5^S_2_ state from which green anti-Stokes emission results. These processes for excitation in the 720–750, 870–920 and 960–1000 nm spectral ranges are interpreted in Figure 8 and are confirmed by the excitation spectrum of up-converted ^5^S_2_ green emission presented in Figure 9. Similar spectra were also reported for other Ho^3+^ activated systems [14].

On the other hand, the energy level diagram in Figure 8 illustrates that in the up-conversion excitation of ^5^S_2_ emission several near-resonant transitions, with participation of low energy phonons, are involved. It can also be observed that energies of GSA and ESA transitions are in close proximity, so it is difficult to indicate one responsible process. We have focused our attention on the 750 nm excitation band, as it was reported to be responsible for the efficient photon avalanche process that occurs in Ho^3+^ activated solids [33].

As results from Figure 8, after 750 nm excitation, the first step is the weak quasi-resonant GSA ^5^I_8_ → ^5^I_4_ absorption, followed by non-radiative de-excitation to the ^5^I_6_ and ^5^I_7_ metastable levels which act as intermediate levels. The next steps are: a strong resonant ESA ^5^I_7_ → ^5^S_2_, ^5^F_4_ and non-resonant ESA ^5^I_6_ → ^5^G_6_, ^5^F_1_ transitions followed by non-radiative de-excitation to the ^5^S_2_, ^5^F_4_ levels from which an intense green emission is obtained.

Calculated GSA and ESA absorption cross section values for transitions involved in the 750 nm excitation process for SrLaGaO_4_ and several other Ho^3+^ activated materials are summarized in Table 5. The cross-sections of the non-resonant transitions from ^5^I_7_ and ^5^I_6_ to the multi-phonon side bands were calculated in the framework of formalism developed by Auzel [34] who showed that the probability for Stokes excitation with respect to an energy gap ΔE to the electronic level is given by:

*W_S_* = *W_S_*(0) *exp*(−α*_S_* Δ*E*) (8)

where *α_S_* is related to the multi-phonon nonradiative decay parameter α*_NR_* by

*α_NR_* = *α_S_* − (*hω*) − 1/*Nln*(*N/S*_0_) (9)

where *N = E/hω* is the average order of the multi-phonon process, *S*_0_ is the Huang–Rhys electron–phonon coupling parameter at 0 K and *hω* is the highest phonon frequency of the host.

**Figure 10:**
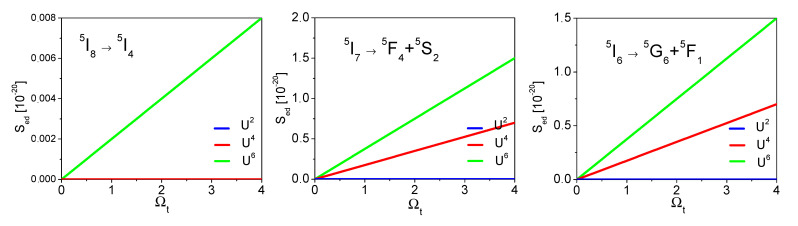
S_ed_ values’ dependence on Ω*_t_* parameters for transitions involved in 750 nm up-conversion excitation.

The values of the integrated cross sections calculated according to Equation (7) are proportional to the line strength of the electric-dipole transition $Sed\left(JJ^{\prime }\right)-\underset{t=2,4,6}{\sum }{\left|U_{JJ’}^{\left(t\right)}\right|}^{2}\Omega_{t}$. Considering the 750 nm excitation conversion to ^5^S_2_ green emission following values of squared matrix elements, |U_λ_|^2^ should be considered. In Figure 10 the S_ed_ values’ dependence on Ω*_t_* is presented.

It thus results that in the considered 750 nm excitation, the ESA (^5^I_7_ → ^5^S_2_ + ^5^F_4_) is the most important process and that for its cross-section to be large, materials with a high value of the Ω_6_ parameter should be chosen. Thus, according to the Judd–Ofelt parameters presented in Table 2, the highest (^5^I_7_ → ^5^S_2_ + ^5^F_4_) ESA cross-sections could be expected for Ho^3+^:LiYF_4_ crystal. This is confirmed by the data reported by Kuck et al. [35]. From Table 6 and Figure 10 it can also be seen that the ^5^I_8_ → ^5^I_4_ GSA is only Ω_6_ dependent.

The ratio between the GSA and ESA cross sections (*β* = GSA/ESA) is an important parameter for establishing the photon avalanche process. It was proposed in [36] that a *β* value lower than 10^−4^ is necessary to expect avalanche up-conversion. Above this limit, the up-conversion emission does not exhibit any pump threshold. Evaluated here *β* = GSA/ESA is about 4 × 10^−3^ and is beyond the limit for photon avalanche up-conversion to occur.

## 4. Conclusions

The spectroscopy and the fluorescence dynamics of SrLaGaO_4_ crystal doped with Ho^3+^ ions have been studied in detail and analyzed. Based on polarized absorption spectra, the Judd–Ofelt intensity parameters were determined and radiative transition probabilities for several Ho^3+^ excited states were calculated. This, together with the measured fluorescence lifetimes, allowed evaluation of the multiphonon relaxation parameters for SLO matrix.

Several ESA mechanisms which generate the green emission from the ^5^S_2_ level in SLO under 750, 900 and 980 nm band excitations have been proposed. The Judd–Ofelt analysis, which is usually performed for transitions from the ground-state manifold, was used to determine the integrated cross-sections of the ESA transitions and to predict the possibility of photon avalanche up-conversion occuring under 750 nm pumping.

## Figures and Tables

**Table 1:** Oscillator strengths (*f_exp_*—measured, *f_theor_*—calculated) of absorption transitions for Ho^3+^ ion in SrLaGaO_4_ crystal.

Transition ^5^I_8_ →	Wavelength λ [nm]	Oscillator Strengths	
		${\overset{¯}{f}}_{exp}$ **[10^−6^]**	${\overset{¯}{f}}_{theor}$ **[10^−6^]**
^5^I_7_	2011	2.19	1.73
^5^I_6_	1169	1.13	1.34
^5^I_5_	902	0.33	0.24
^5^I_4_	748	0.03	0.02
^5^F_5_	647	3.37	2.29
^5^S_2_ + ^5^F_4_	541	3.86	4.04
^5^F_3_	488	0.92	1.58
^5^F_2_	476	0.10	0.92
^3^K_8_	470	0.29	0.84
^5^G_6_ + ^5^F_1_	454	20.7	6.68
RMS = 5.3 × 10^−6^			

**Table 2:** Calculated Judd–Ofelt intensity parameters for SrLaGaO_4_:Ho^3+^ compared with several Ho^3+^ ion activated solids.

Material	Ω_2_ [10^−20^ cm^2^]	Ω_4_ [10^−20^ cm^2^]	Ω_6_ [10^−20^ cm^2^]	Ref.
SrLaGaO_4_ (SLO)	1.25	0.42	1.80	This work
SrLaGa_3_O_7_ (SLG)	2.23	0.85	1.81	[27]
ZBLAN glass	2.46	2.02	1.71	[12]
Y_3_Al_5_O_12_ (YAG)	0.10	2.09	1.72	[28]
LiYF_4_ (YLF)	1.16	2.24	2.09	[29]

**Table 3:** Probabilities of radiative transitions, branching ratios and radiative lifetimes of excited states of Ho^3+^ in SrLaGaO_4_ crystal.

Transition		SrLaGaO_4_:Ho^3+^		
		*A* [1/s]	*β*	*τ_R_*
^3^D_3_ → ^3^G_5_		22	0.011	
	^5^G_5_	70	0.034	
	^3^K_7_	32	0.015	
	^5^G_6_	20	0.009	
	^3^K_8_	821	0.374	
	^5^F_3_	25	0.011	
	^5^F_4_	35	0.016	
	^5^F_5_	11	0.005	
	^5^I_4_	50	0.023	
	^5^I_6_	106	0.141	
	^5^I_7_	794	0.361	
	^5^I_8_	87	0.039	
∑A = 2097			1	477 μs
^5^F_3_ → ^5^F_4_		0	0.000	
	^5^S_2_	0	0.000	
	^5^F_5_	1	0.000	
	^5^I_4_	112	0.024	
	^5^I_5_	50	0.011	
	^5^I_6_	265	0.057	
	^5^I_7_	761	0.165	
	^5^I_8_	3427	0.742	
∑A = 4615			1	217 μs
^5^S_2_ → ^5^F_5_		0	0.000	
	^5^I_4_	64	0.013	
	^5^I_5_	64	0.014	
	^5^I_6_	256	0.054	
	^5^I_7_	1835	0.388	
	^5^I_8_	2511	0.531	
∑A = 4731			1	211 μs
^5^F_5_ → ^5^I_4_		0	0.000	
	^5^I_5_	11	0.004	
	^5^I_6_	140	0.052	
	^5^I_7_	471	0.175	
	^5^I_8_	2068	0.769	
∑A = 2690			1	372 μs
^5^I_4_ → ^5^I_5_		9	0.050	
	^5^I_6_	70	0.371	
	^5^I_7_	90	0.478	
	^5^I_8_	19	0.100	
∑A = 187			1	5.34 ms
^5^I_5_ → ^5^I_6_		9	0.031	
	^5^I_7_	173	0.583	
	^5^I_8_	115	0.386	
∑A = 297			1	3.37 ms
^5^I_6_ → ^5^I_7_		30	0.084	
	^5^I_8_	324	0.916	
∑A = 353			1	2.83 ms
^5^I_7_ → ^5^I_8_		125	1	8.02 ms

**Table 4:** Calculated radiative lifetimes *τ_R_*, lifetimes observed at T = 10 K *τ_obs_*, energy gaps ΔE, and obtained multiphonon transition rates *W_nr_* for excited states of Ho^3+^ in SrLaGaO_4_ crystal.

Manifold	SrLaGaO_4_:Ho^3+^			
	*τ_R_* [µs]	*τ_obs_* [µs]	Δ*E*_*i*(*i*−1)_ [cm^−1^]	*W_nr_* [s^−1^]
^3^D_3_	477	35	2100	26,500
^5^S_2_	211	151	2794	1883
^5^F_5_	372	7	~1950	140,169
^5^I_6_	2833	1200	3426	480
^5^I_7_	8015	4000	4225	125

**Table 5:** Calculated GSA and ESA absorption cross section values for transitions involved in the 750 nm excitation process for SrLaGaO_4_ and several other Ho^3+^ activated materials.

Material	^5^I_8_ → ^5^I_4_	^5^I_7_ → ^5^S_2_ + ^5^F_4_	^5^I_6_ → ^5^G_6_ + ^5^F_1_	Ref.
	*σ* (GSA) [10^−20^ cm^2^]	*σ* (ESA) [10^−20^ cm^2^]	*σ* (ESA) [10^−20^ cm^2^]	
SLO	0.0053	1.242	0.903	This work
SLG	0.0037	1.318	0.116	[27]
ZBLAN	0.0025	1.220	0.942	[12]
YAG	0.0036	1.309	1.202	[28]
YLF	0.0037	1.405	1.080	[29]

**Table 6:** Doubly reduced matrix elements for selected transitions in Ho^3+^ ions.

**|** *U_t_* **|** ^2^	^5^I_8_ → ^5^I_4_	^5^I_7_ → ^5^F_4_	^5^I_7_ → ^5^S_2_	^5^I_7_ → ^5^S_2_ + ^5^F_4_	^5^I_6_ → ^5^G_6_	^5^I_6_ → ^5^F_1_	^5^I_6_ → ^5^G_6_ + ^5^F_1_
|*U^2^*|^2^	0	0	0	0	0.0093	0	0.0093
|*U^4^*|^2^	0	0.1965	0	0.1965	0.0837	0	0.0837
|*U^6^*|^2^	0.0082	0.0349	0.4195	0.4544	0.1094	0.2383	0.3477

## Data Availability

The data presented in this study are available on reasonable request from the corresponding author.
